# Pectin supplement alleviates gut injury potentially through improving gut microbiota community in piglets

**DOI:** 10.3389/fmicb.2022.1069694

**Published:** 2022-12-09

**Authors:** Guoqi Dang, Wenxing Wang, Ruqing Zhong, Weida Wu, Liang Chen, Hongfu Zhang

**Affiliations:** ^1^State Key Laboratory of Animal Nutrition, Institute of Animal Sciences, Chinese Academy of Agricultural Sciences, Beijing, China; ^2^Precision Livestock and Nutrition Unit, Gembloux Agro-Bio Tech, TERRA Teaching and Research Centre, Liège University, Gembloux, Belgium

**Keywords:** pectin, SCFA, gut microbiota, inflammatory responses, pig

## Abstract

As pectin is widely used as a food and feed additive due to its tremendous prebiotic potentials for gut health. Yet, the underlying mechanisms associated with its protective effect remain unclear. Twenty-four piglets (Yorkshire × Landrace, 6.77 ± 0.92 kg) were randomly divided into three groups with eight replicates per treatment: (1) Control group (CON), (2) Lipopolysaccharide-challenged group (LPS), (3) Pectin-LPS group (PECL). Piglets were administrated with LPS or saline on d14 and 21 of the experiment. Piglets in each group were fed with corn-soybean meal diets containing 5% citrus pectin or 5% microcrystalline cellulose. Our result showed that pectin alleviated the morphological damage features by restoring the goblet numbers which the pig induced by LPS in the cecum. Besides, compared with the LPS group, pectin supplementation elevated the mRNA expression of tight junction protein [*Claudin-1*, *Claudin-4*, and *zonula occludens-*1 (*ZO-1*)], mucin (*Muc-2*), and anti-inflammatory cytokines [*interleukin 10* (*IL-10*), and *IL-22*]. Whereas pectin downregulated the expression of proinflammatory cytokines (*IL-1β*, *IL-6*, *IL-18*), *tumor necrosis factor-&alpha* (*TNF-α*), and *NF-κB*. What is more, pectin supplementation also significantly increased the abundance of beneficial bacteria (*Lactobacillus, Clostridium_sensu_stricto_1*, *Blautia*, and *Subdoligranulum*), and significantly reduced the abundance of harmful bacteria, such as *Streptococcus*. Additionally, pectin restored the amount of short-chain fatty acids (SCFAs) after being decreased by LPS (mainly Acetic acid, Propionic acid, and Butyric acid) to alleviate gut injury and improve gut immunity *via* activating relative receptors (*GPR43*, *GPR109*, *AhR*). Mantel test and correlation analysis also revealed associations between intestinal microbiota and intestinal morphology, and intestinal inflammation in piglets. Taken together, dietary pectin supplementation enhances the gut barrier and improves immunity to ameliorate LPS-induced injury by optimizing gut microbiota and their metabolites.

## Introduction

The weaning transition is a critical period for mammalian growth (Holman et al., 2021; Gonzalez-Sole et al., 2022). Due to the inadequate development of immunological and digestive systems (Hughes et al., 2017), they are more susceptible to viruses and harmful bacteria, which can damage intestinal function and cause debilitating diarrhea. However, gut microbial dysbiosis is a major cause of neonatal and post-weaning diarrhea in pigs (Hughes et al., 2017). Trillions of microorganisms are colonized in the mammalian gut (Lynch and Pedersen, 2016). The intestinal flora is a mutually beneficial relationship between the host and the intestinal flora that regulates the body’s food intake and metabolism, serves as a defense against toxins and external antigens, and fosters the growth of the intestinal immune system (Reyman et al., 2019). Very recently, the effect of intestinal microbiota on the host might be mediated by influences on the microbial metabolites (Wan et al., 2021). Acetic acid, propionic acid, and butyric acid are the three of the most representative short-chain fatty acids (SCFAs). The acetic acid in the intestine is mainly produced by the fermentation of *Bifidobacterium* spp. and *Lactobacillus spp*. (Zuniga et al., 2018). It constitutes the highest proportion of short-chain fatty acids produced by intestinal bacterias. Acetic acid regulates the pH level, maintains the homeostasis of the intestinal environment, nourishes beneficial microorganisms, and prevents the invasion of harmful bacteria and opportunistic pathogens (Konda et al., 2020). *Escherichia coli*, *Mycobacterium fragilis* and *Microcystis aeruginosa* are the main propionic acid-producing bacterias. In addition to their functional similarities with acetic acid, propionic acid can also regulate appetite through *PYY* and *GLP-1* (Canfora et al., 2019). Butyric acid is absorbed directly into the colonic epithelium, where it is oxidized to produce butyryl coenzyme and used in the synthesis of ATP. It also has the vital function of maintaining the integrity of the intestinal wall (Dang et al., 2021).

Dietary fiber (DF) is a carbohydrate polymer with more than 10 monomeric units, making it difficult to be hydrolyzed and absorbed by endogenous enzymes in the small intestine (Nevara et al., 2021). According to its solubility, DF is usually divided into two categories: soluble dietary fiber (SDF, e.g., pectin) and insoluble dietary fiber (IDF, e.g., cellulose; Xia et al., 2021). There are many ways for pectin to regulate the host, such as regulating the composition of intestinal microbes, regulating intestinal permeability, and reducing intestinal inflammation. Beyond that, the effect of microbial metabolites (short-chain fatty acids) is a non-negligible regulatory pathway. Pectin is mainly a group of acids heteropolysaccharides consisting of *D*-galacturonic Acids (*D*-Gal-A) linked by α-1,4-glycosidic bonds (Wu et al., 2020). Besides, it also contains neutral sugars, such as L-rhamnose, D-galactose, and D-arabinose. And it is abundant in the peels of citrus, lemon, and grapefruit.

In recent years, the application of microbiology in the analysis of dietary fibers on the intestinal immune factors has extensively increased, but biological meaningful pathways, for instance, the regulatory and metabolic pathways, are still poorly understood. Thus, in this study, lipopolysaccharide (LPS) intraperitoneal injection was used to establish the intestinal injury model of piglets, and the aim of this research was to explore whether pectin supplementation in the diet could alleviate intestinal injury *via* gut microbiota community in piglets.

## Materials and methods

### Animal management, experimental design, and sample collection

All procedures in this study received ethical approval from the Experimental Animal Welfare and Ethical Committee of Institute of Animal Science of Chinese Academy of Agricultural Sciences (IAS2019-37). A total of 24 21-day-old pigs (Yorkshire × Landrace, 6.77 ± 0.92 kg), half male and female piglets in each group, were randomly distributed into three groups with eight replicates per treatment: (1) Control group (CON), (2) LPS-challenged group (LPS), (3) Pectin-LPS group (PECL). The initial body weight and health status of the piglets were no significant difference among the groups in this study. Each group of piglets was fed with corn-soybean meal diets containing 5% citrus pectin (PEC, [with a purity of >81.4%] purchased from Yuzhong Biotech Corporation, Henan, China) or microcrystalline cellulose (MCC [99.5% purity], purchased from Engineering research center of cellulose and its derivatives, Beijing, China) respectively.

The experiment lasted for 28 days which consisted of a pre-starter period (7 days) and a starter period (21 days). During the whole test period, the diets had been formulated to meet the nutritional requirements suggested by NRC (2012) for pigs within the corresponding weight range (Table 1). On day 14 and day 21, piglets from LPS group and PECL group received a simulated bacterial challenge by an intraperitoneal injection of LPS solution (80 μg per kg BW, *E. coli* 0111: B4, Sigma). And meanwhile, piglets in the CON group received an intraperitoneal injection of 200 μl of saline. Previous studies proved that LPS causes serious damage to the intestinal structure and barrier function within 2 to 6 h after injection (Xu et al., 2020). Hence, 3 h after the 2nd injection of LPS or Saline, blood samples were collected from the anterior vena cava before being anaesthetized. After slaughtering, samples of cecal contents and mucosa were obtained and immediately frozen in liquid nitrogen and then transferred to −80°C for further analysis.

**Table 1:** The composition and nutrient content of basal diets (air-dry basis) %.

Ingredients	CON / LPS	PECL
Expanded corn	46.73	46.73
Expanded soybean	10	10.00
Soybean meal	13	13.00
Fish meal	4.5	4.50
Dried whey	10	10.00
Soybean oil	2	2.00
CaHPO_4_	0.5	0.50
NaCl	0.3	0.30
Limestone	0.51	0.51
Choline chloride	0.09	0.09
Lysine	0.8	0.80
Met	0.17	0.17
Thr	0.32	0.32
Trp	0.08	0.08
Sugar	2.5	2.50
Glucose	2.5	2.50
Premix1)	1	1.00
Cellulose	5	
Pectin		5.00
Nutrient levels2)		
GE Mcal/kg	3.94	4.01
Crude protein	18.01	17.90
Ca	0.67	0.67
TP	0.54	0.54
AP	0.39	0.39
Lys	1.51	1.51
Met	0.44	0.44
Met+Cys	0.68	0.68
Thr	0.91	0.91
Trp	0.26	0.26

(1) The premix provides per kg of diet: VA: 18000 IU; VD3: 4500 IU; VE: 22.5 mg; VK3: 4.5 mg; VB1: 4.32 mg; VB2: 12 mg; VB6: 4.86 mg; VB12: 30 μg; Biotin: 480 μg; Folic acid: 1.764 mg; Calcium Pantothenate: 33.12 mg; Nicotinamide: 41.58 mg; Cu: 20 mg; Fe: 140 mg; Zn: 140 mg; Mn: 40 mg; Se: 0.3 mg; I: 0.5 mg.

(2) GE and Crude protein are measured values, while the other nutrient levels are calculated values.

### Counting the number of goblet cells

The cecum segment was fixed in paraformaldehyde, dehydrated, and embedded in paraffin to prepare paraffin sections (section thickness of 5um), and then stained with PAS solution. The number of goblet cells was assessed by random measurement of 10 crypts per section using DS-U3 (Nikon, Japan). For more specific details about this process, we refer to (Tang et al., 2022).

### DNA extraction, real-time PCR analysis

We extract the total RNA from cecal mucosa using the RNeasy Mini Kit (GeneBetter, Beijing, China). The concentration of each RNA sample was quantified using the NanoDrop 2000 (Nanodrop Technologies, Wilmington, DE, USA). The cDNA was transcribed by using the High-Capacity cDNA Archive kit (Takara, Takara Biomedical Technology in Beijing, China). qRT-PCR was conducted with a commercial kit (PerfectStart Green Qpcr SuperMix, Transgen in Beijing, China). The mRNA level of *β-actin* was used as an internal control. The relative genes expression of mRNA of tight junction protein (*Claudin-1*, *Claudin-4, ZO-1*), mucin (*Muc-2*) inflammatory cytokine genes (*IL-1β*, *IL-6*, *IL-8*, *IL-18*, *TNF-α*, *NF-κB*, *IL-10*, and *IL-22*), and immune-related receptors (*GPR41*, *GPR43*, *GPR109*, *AHR*) was detected by qRT-PCR. A single peak was checked to confirm the specificity of each primer (Table 2) set in the melting curves after 40 PCR amplification cycles. (Primer efficiency was checked by using different cDNA concentrations and only primer with mathematical efficiency between 90 and 110% were used). Relative expression of each primer between the control group and treatment group was calculated by 2^-△△Ct^ method, and the values were normalized to the reference house-keeping genes *β-actin*.

**Table 2:** Primers used for real-time quantitative PCR analysis.

Genes	Primers	Promers sequences (5′ to 3′)
β-actin	F	GCGTAGCATTTGCTGCATGA
	R	GCGTGTGTGTAACTAGGGGT
Claudin-1	F	TCGACTCCTTGCTGAATCTG
	R	TTACCATACCTTGCTGTGGC
Claudin-4	F	CAACTGCGTGGATGATGAGA
	R	CCAGGGGATTGTAGAAGTCG
MUC2	F	CGCATGGATGGCTGTTTCTG
	R	ATTGCTCGCAGTTGTTGGTG
ZO-1	F	CTCCAGGCCCTTACCTTTCG
	R	GGGGTAGGGGTCCTTCCTAT
IL-1β	F	GCCAGTCTTCATTGTTCAGGTTT
	R	CCAAGGTCCAGGTTTTGGGT
IL-6	F	TCCAATCTGGGTTCAATCA
	R	TCTTTCCCTTTTGCCTCA
IL-8	F	TACGCATTCCACACCTTTC
	R	GGCAGACCTCTTTTCCATT
IL-10	F	TCGGCCCAGTGAAGAGTTTC
	R	GGAGTTCACGTGCTCCTTGA
IL-18	F	TCCGGATCACTTCCTCTCGT
	R	CCGATTCCAGGTCTTCATCGT
IL-22	F	AGCAAGCGTGAAGGTGCGGTT
	R	GCGGACATCTGGGAGCCCTTT
TNF-α	F	CGTCGCCCACGTTGTAGCCAAT
	R	GCCCATCTGTCGGCACCACC
NF-κB	F	AGTACCCTGAGGCTATAACTCGC
	R	TCCGCAATGGAGGAGAAGTC
GPR41	F	GTCTGTGCCCTCATGGGTTT
	R	GACGTTCATACCTTCGGCCT
GPR43	F	CCTGACGCTGGCAGACCT
	R	GCTGCTGTAGAAGCCGAAACC
GPR109	F	AGCCATCATCTCCTGCCTCCTG
	R	ATCATGCCAGCGGAAGGTATTGC
AhR	F	CATGCTTTGGTCTTTTATGC
	R	TTCCCTTTCTTTTTCTGTCC

### 16S rRNA gene sequencing

Total bacterial DNA was extracted from the intestinal chyme and mucosa using the EZNATM Soil DNA kit (D5625-02, Omega Bio- Tek Inc., Norcross, GA, USA) according to the instructions of the manufacturer. The V3-V4 hypervariable regions of the bacterial 16S rDNA were amplified by a two-step PCR method using primers 338F (5′-ACTCCTRCGGGAGGCAGCAG-3′) and 806R (5′-GGACTACCVGGGTATCTAAT-3′) with unique 8-bp barcodes to facilitate multiplexing, and sequencing was carried out with an Illumina sequencing platform using Miseq PE300 (Illumina, San Diego, USA) according to the standard protocols by Majorbio Bio-Pharm Technology Co. Ltd. (Shanghai, China).

The raw 16S rRNA gene sequencing reads were demultiplexed, quality-filtered by fastp version 0.20.0 (Chen et al., 2018), and merged by FLASH version 1.2.7 (Magoc and Salzberg, 2011). Operational taxonomic units (OTUs) with 97% similarity cutoff (Edgar, 2013) were clustered using UPARSE version 7.1 [3], and chimeric sequences were identified and removed. The taxonomy of each OTU representative sequence was analyzed by RDP Classifier version 2.2 (Wang et al., 2007) against the 16S rRNA database using a confidence threshold of 70% (PRJNA889391).

### Western blot

Tissue total protein was extracted in a RIPA lysis buffer on ice. Protein content was quantified using the BCA protein assay kit (Cat# 23225, Thermo, Waltham, MA, USA). A total of 30 μg protein was loaded per lane and boiled for 15 min before separation by 10% SDS-PAGE gel. The SDS-PAGE gel result was transferred onto a polyvinylidene difluoride membrane under 90 V for 1.5 h using the wet transfer method. Then the membranes were incubated in 5% skimmed milk for 2 h at room temperature for blotting. After incubation with a primary antibody of Occlaudin (Thermo Fisher Scientific Inc., MA, USA, #40–4,700, 1:500)), Claudin-1 (Thermo Fisher Scientific Inc., MA, USA, #51–9,000, 1:500), and β-actin (Proteintech, Chicago, USA, #20536-1-AP, 1:1,000) overnight at 4°C, the membrane was washed with TBST buffer and incubated with the HRP-labeled goat anti-rabbit/mouse second antibody (Abcam, Cambridge, UK, 10#ab6721, 1:5,000) for 40 min at room temperature. Protein blots were visualized using SuperSignal® West Femto Maximum Sensitivity Substrate (Cat # 34094, Thermo) and a gel imaging system (Tanon Science & Technology Co., Ltd., China). Band density was quantified by the Image J 10.0 software and normalized to β-Actin.

### Metabolite determination of gut microbiota

Quantification of Cecum Short-Chain Fatty Acids (SCFAs) in pig cecal contents were measured as described in our previous report (Tang et al., 2021). Briefly, cecal contents (200.0 mg) were thoroughly mixed with ultrapure water at a ratio of 1:9, then shocked for 30 min to mix evenly, and then incubated at 4°C overnight and then centrifuged at 10000 g for 10 min. After this, 0.9 milliliters of the supernatant were mixed with 0.1 ml of metaphosphoric acid (25% (v/v)) and kept for 3 h. The sample was then cleared by centrifugation at 10,000 g for 10 min and passed through a 0.45-μm Milled-LG filter (Jinteng, Tianjin, China) and subjected to SCFA analysis.

### Data analysis and statistical test

Data on cytokines, bacterial α-diversity indices (Chao1, and Shannon), microbial metabolites (SCFAs), and gene expression were analyzed by the Tukey–Kramer test and the Duncan multiple comparison method (JMP version 10.0, SAS Institute, Inc., Cary, NC, USA). Significance is presented as *p < 0.05, **p < 0.01, and ***p < 0.001. In addition, the correlation analysis was performed using the Mental test and Spearman’s correlation (R package “pheatmap”).

## Results

### Pectin supplementation affected cecal permeability and histomorphology

**Figure. 1:**
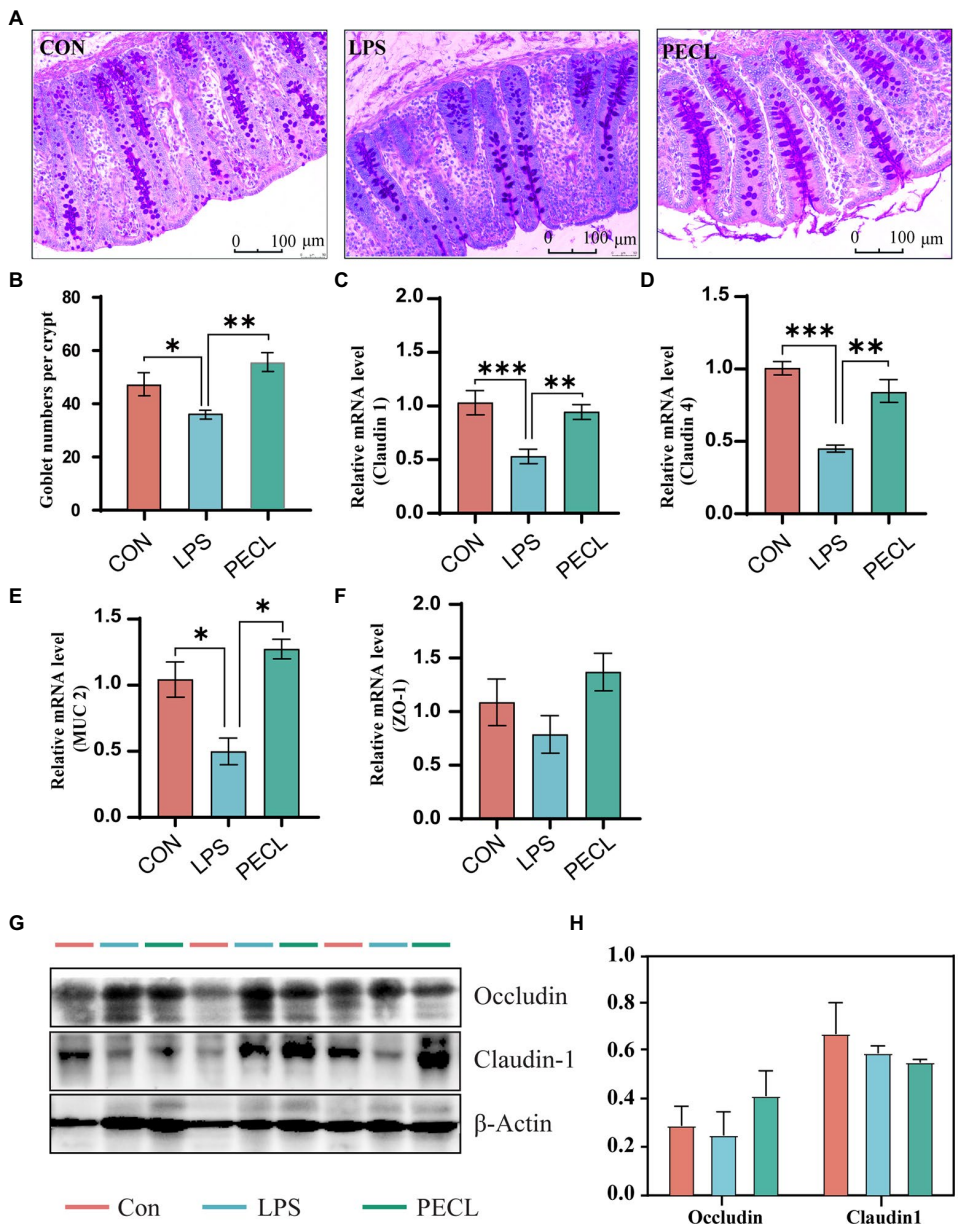
Effects of dietary supplemented with pectin on gut morphology of LPS challenged piglets. **(A)** Representative PAS-stained cecum sections. **(B)** Goblet number. The fold change in mRNA expression relative to β-actin for **(C)** *Claudin-1*, **(D)** *Claudin-4*, **(E)** *ZO-1*, **(F)** *Muc-2* is shown. And **(G,H)** Western blot analysis of Occludin and Claudin1. Signification is presented as *p < 0.05, **p < 0.01, and ***p < 0.001; data are presented as the mean ± SE (mRNA, *n *= 8; WB, *n* = 3).

The piglets challenged with LPS showed signs of diarrhea, fever, and cough. To observe morphological and histological changes in the cecum, we performed PAS staining. It was apparent from Figure 1 that LPS decreased the number of goblet cells compared with that in the CON group (*p* < 0.05). Addition of pectin significantly increased the number of goblet numbers when compared with the LPS group (Figures 1A,B; *p* < 0.01). Furthermore, LPS significantly decreased the mRNA expression levels of tight junction protein [*Claudin 1*, Figure 1C, (*p* < 0.001), *Claudin 4*, Figure 1D, (*p* < 0.001)], pectin supplementation notably increased the mRNA levels of *Claudin 1* (*p* < 0.01), *Claudin 4* (*p* < 0.01), compared with LPS group (Figure 1C,D). Similarly, piglets challenged with LPS significantly reduced the mRNA expression of *Muc2*, and it was restored after the addition of pectin in PEC group (*p* < 0.05). Although WB data did not reach the significant levels (Figures 1G,H). Accordingly, pectin supplementation markedly restored the intestine damage in piglets due to LPS stress.

### Pectin modified inflammatory cytokine gene expression in the cecum

**Figure. 2:**
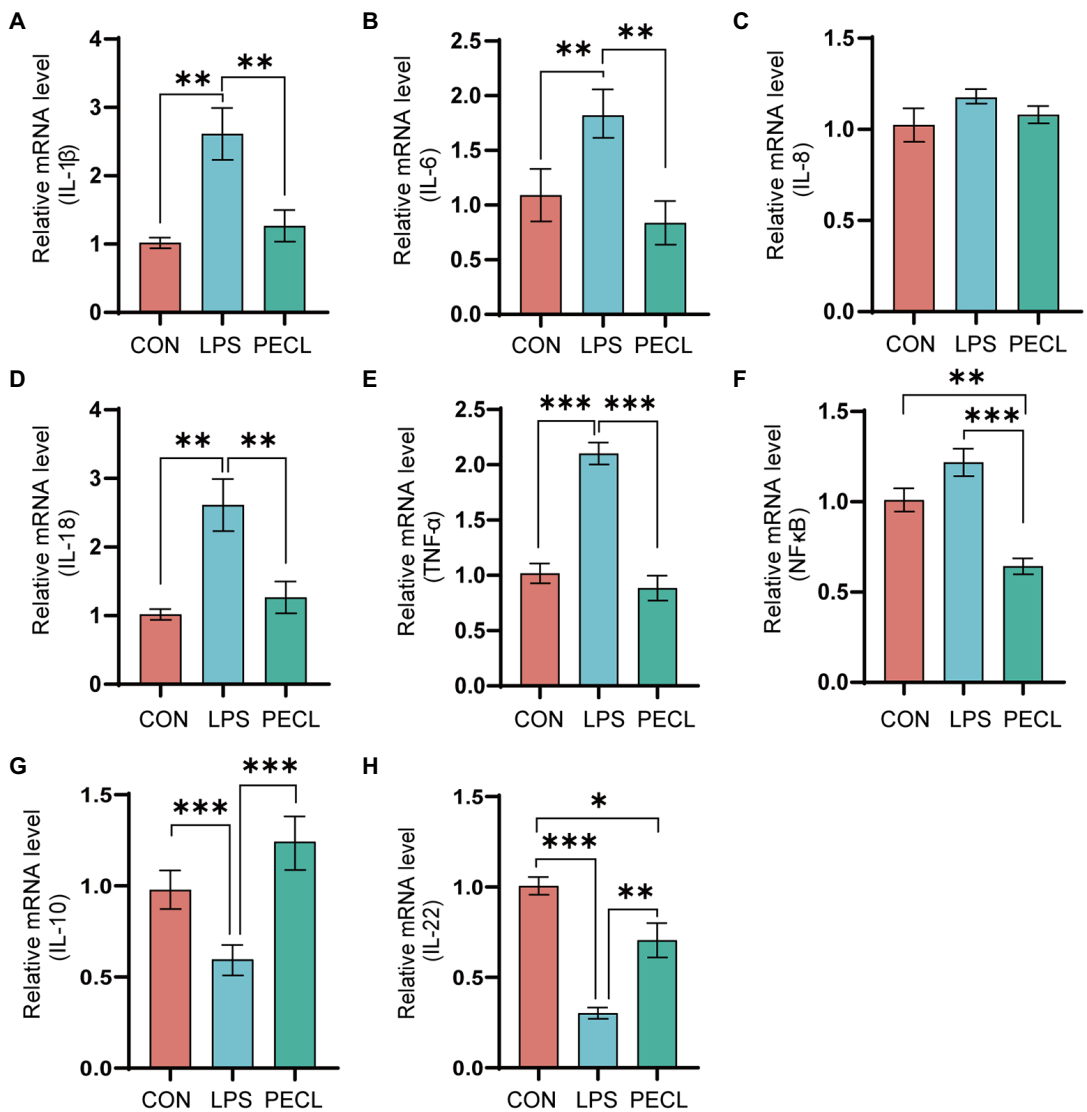
Effects of dietary pectin on inflammatory cytokines in cecum. The fold change in mRNA expression relative to β-actin for **(A)** *IL-1β*, **(B)** *IL-6*, **(C)** *IL-8*, **(D)**, *IL-18*, **(E)** *TNF-α*, **(F)**, *NF-κb*, **(G)** *IL-10*, **(H)** *IL-22* is shown. Signification is presented as *p < 0.05, **p < 0.01, and ***p < 0.001; data are presented as the mean ± SE (*n* = 8).

As shown in Figure 2, piglets in the LPS group had higher expression levels of *IL-1β* (*p* < 0.01), *IL-6* (*p* < 0.01), *IL-18* (*p* < 0.01), *TNF-α* (*p* < 0.001), and *NF-κB* (Figures 2A,B,D,E), and lower expression levels of *IL-10* (*p* < 0.001), *IL-22* (*p* < 0.001; Figures 2G,H) than piglets in CON group. After adding pectin, the mRNA expression levels of pro-inflammatory was significantly reduced, including *IL-1β* (Figure 2A; *p* < 0.01), *IL-6* (Figure 2B; *p* < 0.01), *IL-18* (Figure 2D; *p* < 0.001), *TNF-α* (Figure 2E; *p* < 0.001), and *NF-κB* (Figure 2F; *p* < 0.001), and restored the levels of the anti-inflammatory *IL-10* (Figure 2G; *p* < 0.001) and *IL-22* (Figure 2H; *p* < 0.01).

### Pectin addition altered the composition of cecal microbiota

**Figure 3:**
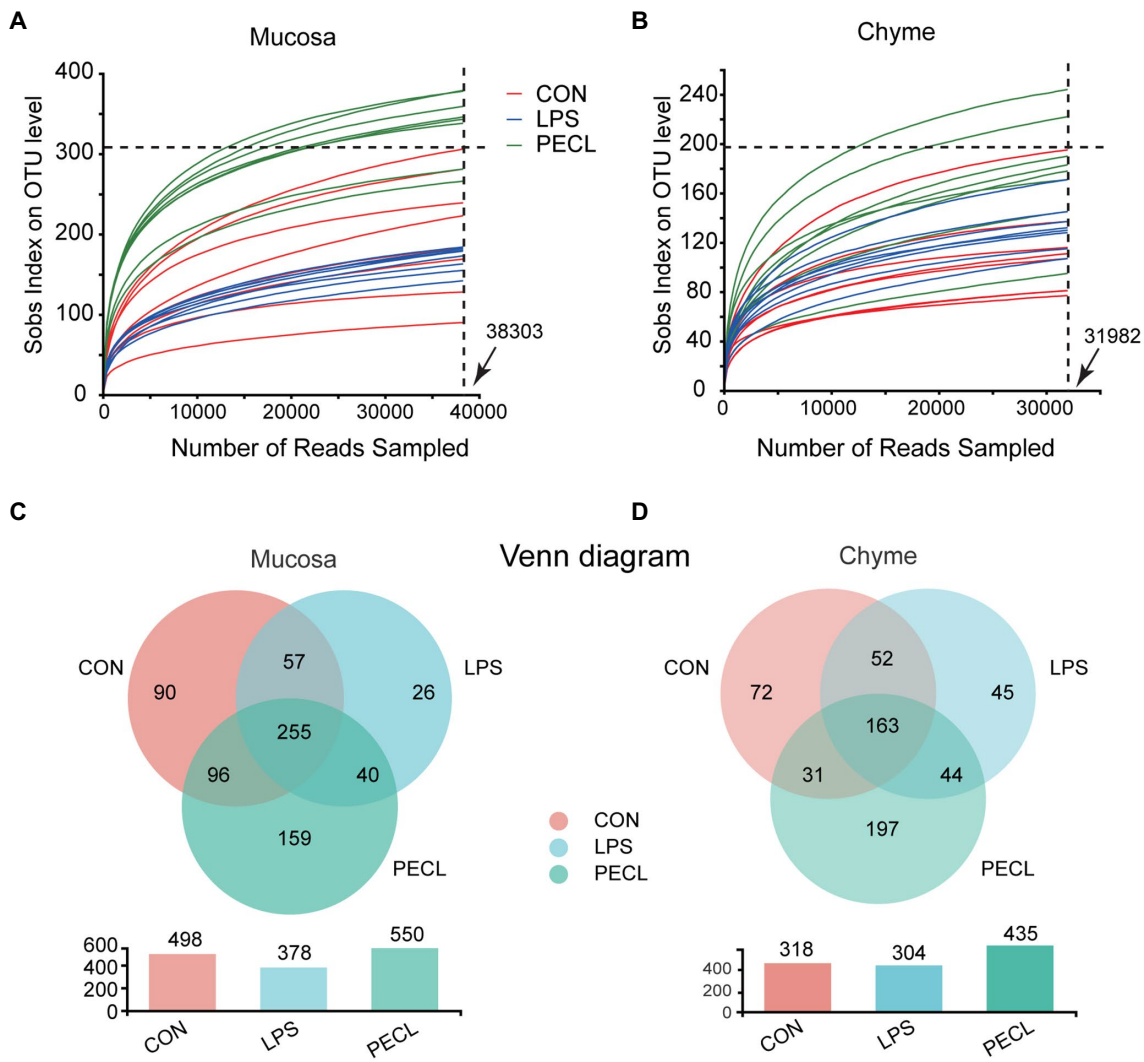
Effects of Pectin on Gut Microbiota diversity. **(A,B)** The rarefaction curves of sobs index on OUT levels of mucosa and chyme in piglets. **(C,D)** Venn diagrams showing the overlap of the OTUs identified in intestinal chyme microbiota and mucosa. Signification is presented as *p < 0.05, **p < 0.01, and ***p < 0.001; data are presented as the mean ± SE (*n* = 8).

We profiled the composition and structure of the microbial communities in both mucosa and chyme of cecum using 16S rRNA amplicon sequencing. Rarefaction curves approached asymptotes across all samples, implying that the sequencing depth was sufficient to cover almost all microbes in the samples (Figures 3A,B). The Venn diagram in the cecal mucosa showed that piglets in the CON, LPS, and PECL groups contained 255 same OTUs and 90, 26, and 159 unique OTUs, respectively (Figure 3C). When it comes to cecal chyme, there were 163 common OTUs between these three groups. Meantime, the CON, LPS, and PECL groups contained individual 72, 45, and 197 OTUs, respectively (Figure 3D).

**Figure 4:**
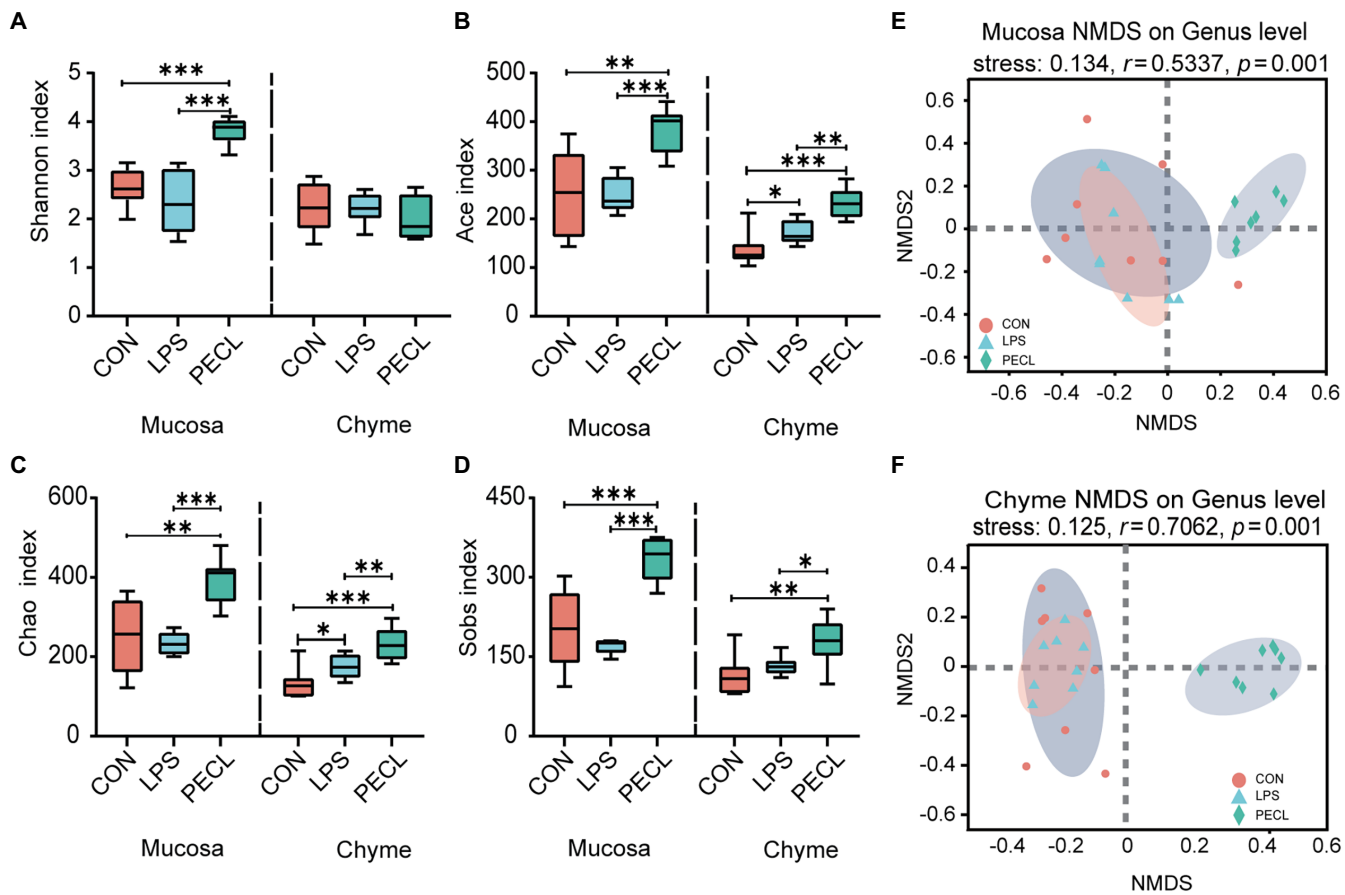
Structure and diversity of intestinal microflora of piglets in different groups. The alpha diversity indices observed species, **(A)** Shannon, **(B)** Ace, **(C)** Chao, and **(D)** Sobs among the three groups. NMDS plot of the chyme microbiota **(E)** and mucosa microbiota **(F)** based on weighted UniFrac distance.

And after quality-filtered and merged, 723 OTUs in mucosa and 704 OTUs in chyme were obtained, respectively. In mucosa, the α-diversity was significantly higher in PECL than in CON and LPS group, while without any difference between CON and LPS group (Figures 4A–D). Similarly, the α-diversity of chyme in PECL was higher than that in other two groups (Figures 4B–D). As for beta diversity result clearly showed dissimilarity among the communities in mucosal and chyme bacteria (Figures 4E,F).

**Figure 5:**
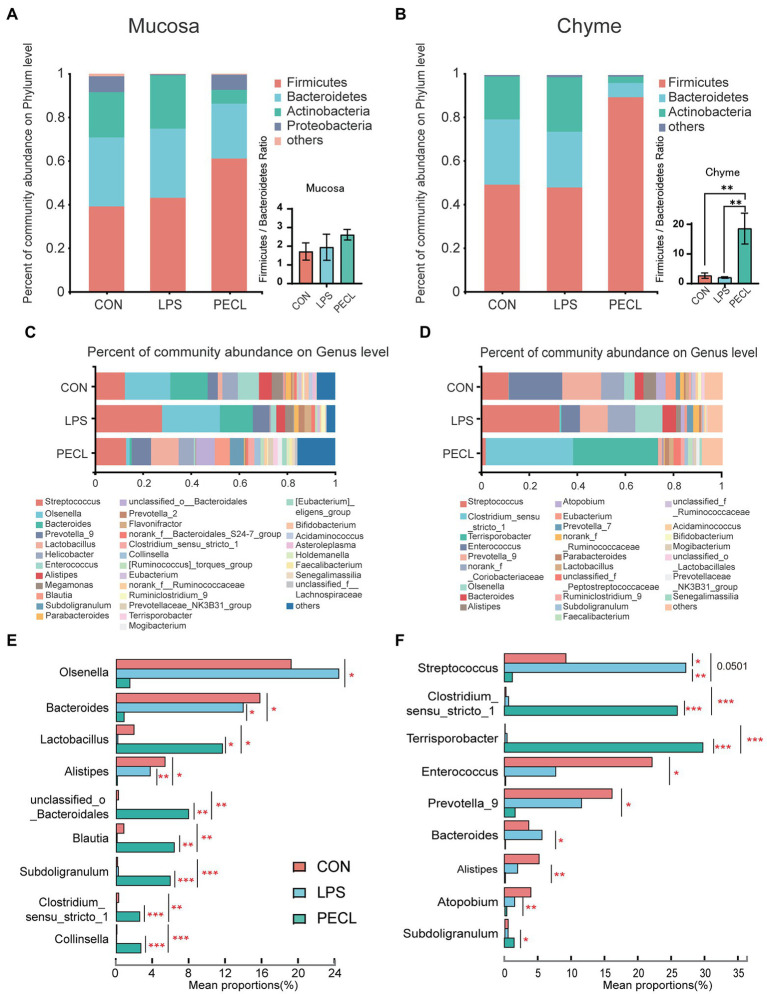
Pectin altered the cecum microbiota in LPS-challenged piglets. Structure comparison of intestinal microbiota in **(A)** mucosa and **(B)** chyme between CON, LPS and PECL groups at Phylum level and Genus level **(C,D)**. Kruskal–Wallis *H* test bar plot shows the changes in the intestinal microbiota in **(E)** mucosa and **(F)** chyme of piglets in different groups at the genus level. Signification is presented as *p < 0.05, **p < 0.01, and ***p < 0.001; data are presented as the mean ± SE (*n* = 8).

As for the microbial composition in mucosa between these three groups. At the phylum, the results showed that *Firmicutes*, *Bacteroides*, *Actinobacteria*, as well as *Proteobactere* were the four main bacterial genera phylum levels in cecum chyme (Figure 5A). Meanwhile, the abundance of *Firmicutes* was higher than that in other groups and decreased the abundance of *Actinobacteria*. As for bacteria genera in genus level of the mucosa, the composition was more complex. The main bacteria genera in the CON group and LPS group were *Streptococcus Olsenella* and *Bacteroides*. These three dominant bacteria accounted for more than 50 and 70% of the intestinal flora, respectively, in the CON group and LPS group, whereas it decreased in the PECL group (Figure 5C).

In cecal chyme, the predominant bacterial communities in CON groups and LPS were *Firmicutes*, *Bacteroides*, and *Actinobacteria* in cecal chyme (Figure 5B). Piglets treated with LPS which fed pectin led to a significant increase in *Firmicutes* as well as a clear decrease in that of *Bacteroidetes* and *Actinobacteria*. Moreover, the ratio of *Firmicutes* / *Bacteroidetes* was significantly higher in the PECL group compared with the CON and LPS group (*p* < 0.01). As for the community abundance on genus level, *Streptococcus*, *Enterococcus*, and *Prevotella_9* were important bacterial genera in the CON group, while the LPS group showed enhanced relative abundance of *streptococcus* and decreased relative abundance of *Enterococcus* and *Prevotella*. And in the PECL group, the abundance of *Streptococcus* was dramatically decreased.

Oppositely, the abundance of *Clostridium* and *Terrisporobacter* was increased (Figure 5D). The differences in abundance between CON, LPS, and PECL groups at the genus level were calculated using the Kruskal-Wallis with FDR correlation. Figures 5E,F listed the top 9 different species (Figures 5E,F). In mucosa, LPS challenge, compared with the CON group, appeared to have some effect on the relative abundance of *Olsenella*, *Bacteroides*, *Lactobacillus*, *Alistipes*, *unclassified_o_Bacteroidales, Blautia*, *Clostridium_sensu_stricto_1*, but the difference did not reach statistical significance. However, in PECL group, the relative abundance of *Olsenella*, *Bacteroides*, and *Alistipes* was decreased, *Lactobacillus*, *unclassified_o_Bacteroidales*, *Blautia*, *Subdoligranulum*, *Clostridium_sensu_stricto_1*, and *Collinsella* were increased compared with LPS group (Figure 5E; *p* < 0.05). And in chyme, the relative abundance of *Streptococcus* was higher due to LPS challenged (*p* < 0.05), whereas *Enterococcus*, *Prevotella_9*, *Alistipes*, and *Atopobium* were decreased, compared with the CON group. After being treated with pectin, the relative abundance of *Clostridium_sensu_stricto_1*, *Terrisporobacter* and *Subdoligranulum* were increased (*p* < 0.05), *Streptococcus*, *Enterococcus*, *Prevotella_9*, *Bacteroides*, *Alistipes*, and *Atopobium* were decreased (Figure 5F; *p* < 0.05).

### Pectin promoted mRNA expression of metabolite-associated receptors in the cecum

**Figure 6:**
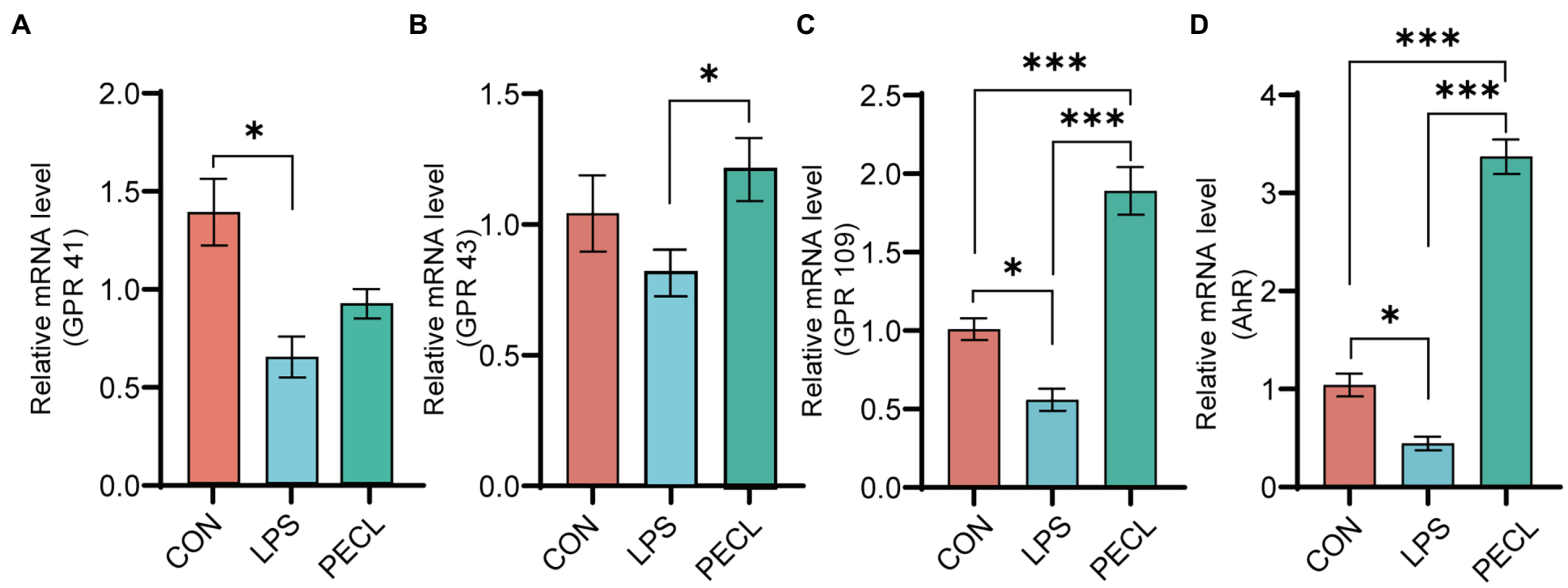
Relative mRNA expression of receptors in cecal mucosa. **(A)** GPR41, **(B)** GPR43, **(C)** GPR109, **(D)** AHR. Signification is presented as *p < 0.05, **p < 0.01, and ***p < 0.001; data are presented as the mean ± SE.

For the expression of metabolite-associated receptors, the data showed in Figure 6. Piglets challenged with LPS decreased the mRNA expression of *GPR41* (*p* < 0.05; Figure 6A), *GPR43* (Figure 6B), *GPR109* (*p* < 0.05; Figure 6C) and *AhR* (*p* < 0.05; Figure 6D). In PECL group, the decreased mRNA expression levels of *GPR43* (*p* < 0.05; Figure 6B)*, GPR109* (*p* < 0.001; Figure 6C) and *AhR* (*p* < 0.001; Figure 6D) were restored by pectin supplementation. Even more, the expression of *GPR109* and *AhR* were notably higher than that in the CON group.

### Addition of pectin increased the concentration of short-chain fatty acids in cecal contents

**Figure 7:**
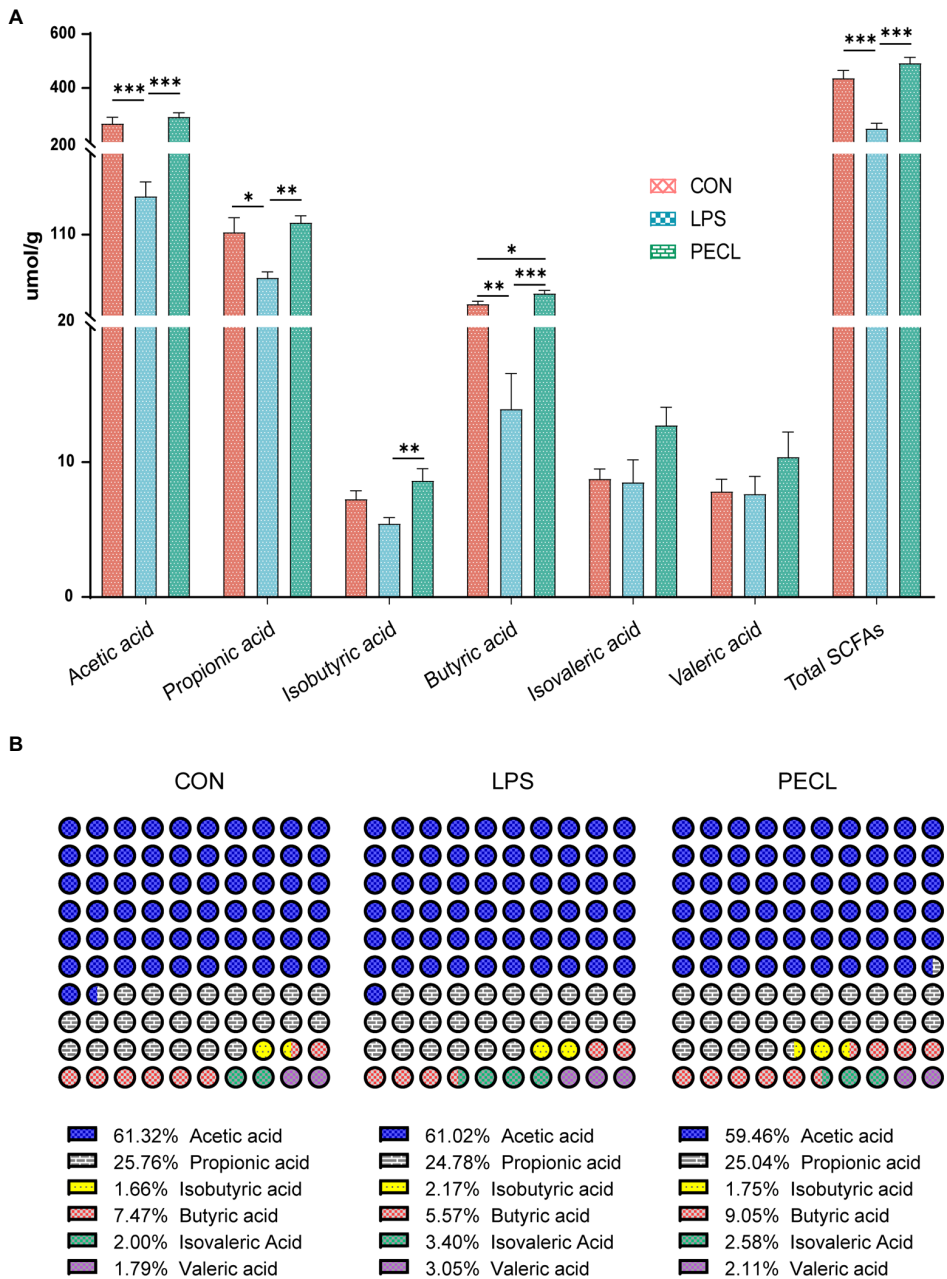
Effects of Pectin on SCFA concentrations **(A)** and compositional proportion **(B)** in the cecum of pigs. Signification is presented as *p < 0.05, **p < 0.01, and ***p < 0.001; data are presented as the mean ± SE.

Among the groups, LPS challenge reduced the concentration of Acetic acid, Propionic acid, Butyric acid, and total SCFA compared with the CON group (Figures 7A; *p* < 0.05). Pectin supplementation markedly restored the levels of Acetic acid, Propionic acid, Isobutyric acid, Butyric acid, and total SCFA (*p* < 0.01), and the concentration of Isovaleric acid tended to increase.

Beyond this, the SCFA composition also varied greatly (Figure 7B). LPS decreased the percentage of propionic acid and butyric acid, whereas increased the percentage of isobutyric acid, isovaleric acid, and Valeric acid. Pectin supplementation restored those shifts, especially in butyric acid. These results demonstrated that supplementation of pectin changed not only the levels of SCFA after being challenged with LPS but also changed the composition ratio of SCFA.

### Effects of dietary pectin supplementation on microbiota-metabolites correlation

**Figure 8:**
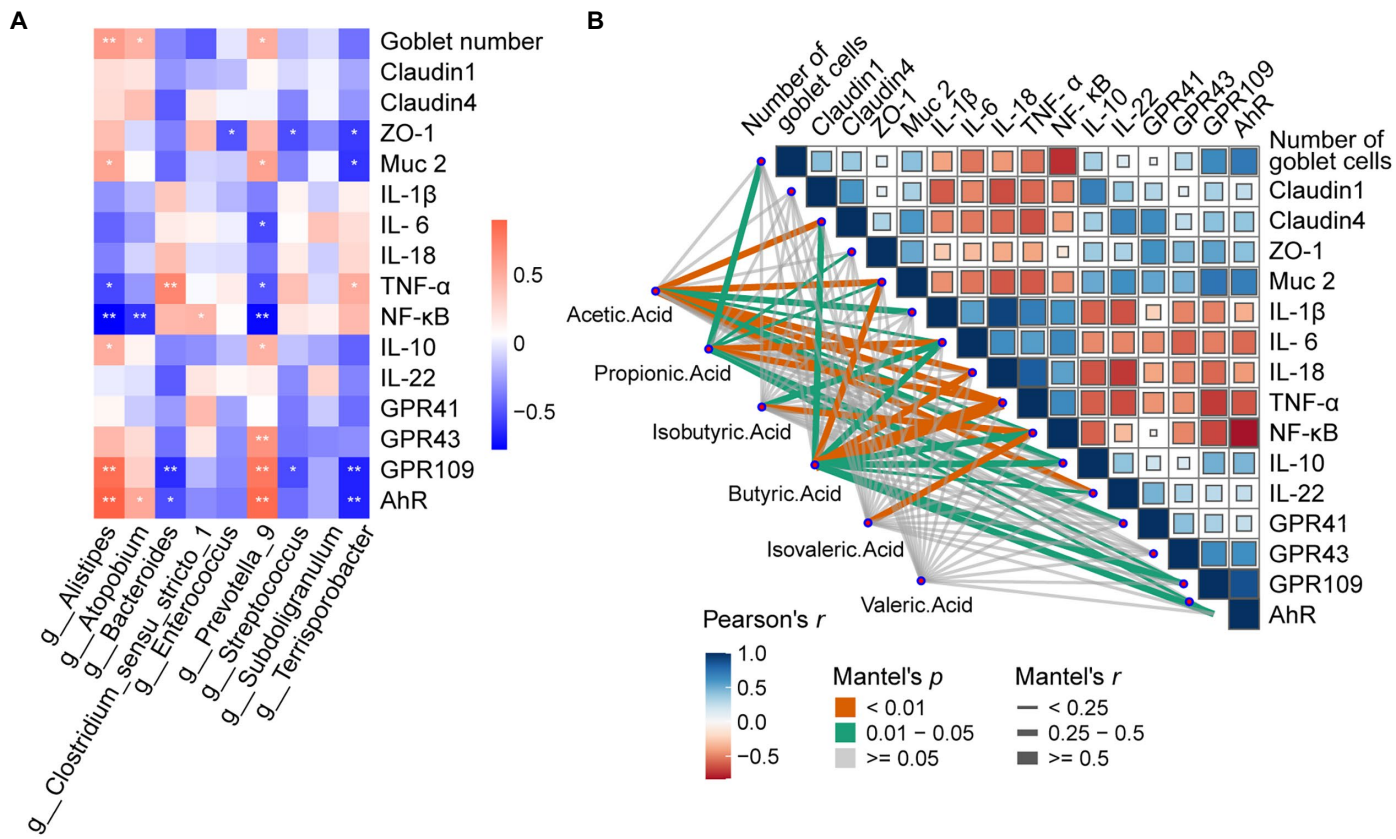
Effects of dietary Pectin supplementation on Microbiota-Metabolites Correlation. **(A)** Spearman’s correlation matrix of Short-chain fatty acids (SCFA), and relative microorganisms in piglets. The direction of ellipses represents positive or negative correlations, and the width of ellipses represents the strength of correlation (narrow ellipse = stronger correlation). Signification is presented as *p < 0.05, **p < 0.01, data are presented as the mean ± SE **(B)** Pairwise comparisons of cecal genera are shown with a color gradient denoting Spearman’s correlation coefficient. Short-Chain Fatty acids were related to other relevant indicators by partial Spearman tests. Edge width corresponds to the Partial Spearman’s *r* statistic for the corresponding distance correlations and edge color denotes the statistical significance.

To find the correlation between the chyme intestinal microbiota and cecal parameters in piglets, spearman’s correlation analysis was carried out based on experimental parameters. As shown in Figure 8A, *g_Terrisporobacter* was positively correlated with Goblet numbers, *Muc-2*, *GPR109*, and *AhR*, but negatively correlated with *TNF-α*, and *NF-κB* (*p* < 0.05). For *g_Subdoligranulum*, Goblet numbers and *AhR* were positively but *NF-κB* was negatively related with it (*p* < 0.05). *g_Streptococcus* was positively correlated with *TNF-α* and negatively correlated with *GPR109*, and *AhR* (*p* < 0.05)*. g_Prevotella_9* was notably positively with *NF-κB* (*p* < 0.05)*. g_Enterococcus* and *g_Bacteroides* had a negative correlation with *ZO-1* (*p* < 0.05)*. g_Clostridium_sensu_stricto_1* was positively correlated with the number of Goblet cells, *Muc-2*, *IL-10*, *GPR43, GPR109*, and *AhR* (*p* < 0.05), but negatively correlated with *IL-6*, *TNF-α, NF-κB* (*p* < 0.05)*. g_Alistipes* was positively correlated with *TNF-α* and negatively related with *ZO-1*, *Muc-2*, *GPR109*, and *AhR* (*p* < 0.05).

Mantel tests were performed to detect the correlation between SCFAs and cytokines in cecal mucosa (Figure 8B). The mantel correlation analysis demonstrated that a significant correlation was observed between *Claudin-4*, *Muc-2*, *IL-1β, IL-6, IL-18, TNF-α*, *NF-κB*, *IL-10*, *IL-22*, and Acetic acid (Mental’s *r >* 0.25, *p <* 0.05). We also found Propionic acid had a powerful relationship with Goblet number, *ZO-1*, *Muc-2*, *IL-6*, *TNF-α*, *GPR41*, *GPR109*, and *AhR*; Isoburic acid had a significant relationship with *IL-6*, and *NF-κB*; Butyric acid had dramatically correlations with *Claudin-4*, *Muc-2, TNF-α*, *NF-κB*, *IL-10*, *IL-22, GPR109*, and *AhR*; Isovaleric acid was associated with *NF-κB*. Thus, SCFAs were closely associated with the goblet cells, tight junction protein, inflammatory cytokines, mucus, and metabolite-related receptors.

## Discussion

Weaning is considered as the most critical period during pig production because of its enormous negative impact on health status and performance (Wang et al., 2018). LPS intraperitoneal injection is widely used in animal studies to establish intestinal injury models to simulate diarrheal pigs in weaning period. Lipopolysaccharide (LPS) intraperitoneal injection is widely used in animal studies to establish intestinal injury models (Wen et al., 2022). Extensive studies showed that LPS could produce a plethora of inflammatory cytokines and damage the intestinal epithelial structure of piglets, resulting in reduced feed intake and diarrhea (He et al., 2019). Besides, recent studies also showed that intestinal injury increases intestinal permeability and bacterial translation (Borisova et al., 2020; Sharma et al., 2020).

Over the years, numerous studies have shown the beneficial effects of pectin and its potential to regulate the inflammatory response (Li et al., 2020), cholesterol (Zofou et al., 2019), and blood glucose (Carvalho et al., 2019). Previous study in our laboratory showed that dietary supplementation of pectin could enhance intestinal barrier function (Wen et al., 2022), increase the expression of *Claudin-4* and *Muc-2* in the cecum of piglets (Wu et al., 2020), and abolish the abnormal expression of *ZO-1* and *Occludin* caused in the acute pancreatitis model (Xiong et al., 2021). In this study, pectin supplementation could improve gut barrier function to alleviate LPS-induced intestinal injury in the cecum by increasing the number of goblet cells, and improving the expression of tight junction proteins and *Muc-2*. Thus, pectin supplementation improves the function of the intestinal barrier and reduces gut inflammation in the pig model.

Cytokines can dynamically regulate the intestinal barrier. The pro-inflammatory factors *IL-1β*, *IL-6* and *TNF-α* can increase intestinal epithelial permeability, induce the pathological opening of the intestinal tight junction barrier, and mediate the inflammatory response. Meanwhile, the anti-inflammatory factors *IL-10* and *IL-22* can maintain homeostasis in the intestine (Xia et al., 2021; Yu et al., 2021). Research showed that mice fed dietary fiber could reduce the concentration of pro-inflammatory cytokines, including *TNF-α* and *IL-6*, while increasing the concentrations of *IL-10* in sera (Zhang et al., 2018). Besides, Chen et al. found that insoluble fiber supplementation decrease the gene expression levels of *IL-1β* and *TNF-α* (Chen et al., 2020). Again, remarkably similar results were obtained by Sun et al. (2021) (Sun et al., 2021). In consistence with previous studies, our results showed that pectin supplementation decreased the mRNA expression levels of *IL-1β*, *IL-6* and *TNF-α* after being increased by LPS. Therefore, pectin may exert an anti-inflammatory effect by regulating the expression levels of cytokines.

Pigs have a developed cecum, which gives piglets a larger relative population of gut microbes and stronger capacity for carbohydrate fermentation. Moreover, when the diversity, constitution, and functions of the gut microbiota are disturbed, the imbalance of the microbiota affects the intestinal immune system *via* metabolite signals or microbial composition (Huang et al., 2020; Lee and Chang, 2021). Recent research showed that the action of dietary fiber in gut disease caused by intestinal microbiome disorders is priceless (Guan et al., 2021; Hua et al., 2021; Usuda et al., 2021). Thus, we assessed the gut microbiota diversity in the cecum mucosa and chyme by 16 s rRNA sequencing of microorganisms. The results revealed that LPS treatment did not exert many differences on the α-diversity (Shannon, Ace, Chao, Sobs). After fed with pectin, the Ace, Chao, and Sobs indexes were boosted. What is more, we found that dietary supplementation with pectin optimized the composition of the intestinal chyme and mucosa microbiota of the LPS challenged including increasing the ratio of *Firmicutes* / *Bacteroides* and the abundance of *Firmicutes*. These observations suggest that the protective effect of pectin is apparently realized by alerting the microbiota. Specifically, at the genus level, we found that the addition of pectin increased the relative abundances of *Clostridium sensu_strict_1*, *Terrisporobacter*, *unclassified_f_ Peptostreptococcaceae*, *Subdoligranulum*, and *Faecalibacterium*. *Clostridium_sensu_stricto_1* and *Terrisporobacter* may directly ferment polysaccharides to SCFAs (Niu et al., 2015; Lu et al., 2021). Besides, *Faecalibacterium* and *Subdoligranulum* are also the major butyrate and Lactic producers in the hindgut (Holmstrom et al., 2004; Bjerrum et al., 2006; Louis and Flint, 2009) which echoes the above that SCFAs were rich in cecal chyme.

At the genus level in the mucosa, *Olsenella* was notably decreased in the pectin supplementation group, which is a propionate-producing bacteria (Adamberg et al., 2018). It has also been confirmed that the percentage of propionate in the pectin group is lower than that in other groups. *Lactobacillus* is an important probiotic which is capable of metabolizing and producing bioactive substances, strengthening the intestinal mucosal barrier, reducing endotoxins, and regulating the body’s immunity (Sarkar and Mandal, 2016). Besides, *Alistipes*, *Blautia*, *Subdoligranulum* as well as *Collinsella* are all microorganisms associated with SCFA producers (Zhou et al., 2017; Gavin et al., 2018; Qin et al., 2019; Das et al., 2021; Kim et al., 2021). Among them, *Alistipes* is a well-known butyric acid producer. *Blautia* is another most abundant member of gut microbiota responsible for the production of butyric acid and acetic acid, which are associated with the improvement in glucose metabolism (Kiros et al., 2019) and the decrease in obesity *via* G-protein coupled receptors 41 and 43 (Ozato et al., 2019). Consistent with the findings of the former study, SCFAs production-related bacteria were significantly higher in the fermented dietary fiber treatment group. This suggests that at least part of the pathway of pectin mitigation of LPS stress is *via* the SCFAs pathway.

Metabolites derived from microbiota have been proven to alter the host’s metabolism and intestinal health (Wu et al., 2021). SCFAs, also known as volatile fatty acids, play an essential role in the storage of energy and the regulation of osmolality in the body. In addition, they are involved in maintaining the function of the intestinal cells, regulating the intestinal immune response, and reducing various inflammatory diseases (den Besten et al., 2013; Koh et al., 2016; Dang et al., 2021). In this study, the supplementation of dietary fiber significantly enhanced the concentration of short-chain fatty acids other than isovaleric acid in the contents of the cecum.

SCFAs have been shown to repair intestinal mucosa and reduce intestinal inflammation by activating GPRs and inhibiting histone deacetylases (HDAC) and downregulating the expression of pro-inflammatory cytokines (Tang et al., 2022). *GPR41*, expressed in gut and adipose tissue, is activated equally by propionate and butyrate (Horiuchi et al., 2020), whereas *GPR43* is more responsive to acetate and propionate than to butyrate. Besides, both *GPR109* and *AhR* can only be activated by butyrate(Dang et al., 2021). In this study, LPS decreased the expression of *GPR41*, *GPR43*, *GPR109*, and *AhR*. And pectin increased the expression of *GPR43*, *109*, and *AhR*, which might be because of the restores of acetate, and propionate in the gut that was reduced by LPS challenge. SCFA further activates *GPR43*, *GPR109*, and *AhR* receptors on the surface of lymphocytes in the cecal mucosa, thereby promoting the host’s immunity and protecting intestinal health.

To better understand the correlations, spearman’s correlation analysis was conducted between the 16sRNA sequencing analysis identified several genera associated with pectin intake, and some of these genera were correlated with levels of Histomorphology, cytokines, and receptors. In a previous study, *Alistipes* was positively correlated with *GPR109*, *AhR* (He et al., 2022). An increased proportion of *Alistipes* may associated with higher levels of, which is identical with our results. Besides, *Prevotella_9* is also a well-known beneficial bacteria (Wang et al., 2019), it showed a positive correlation with *AhR*, *GPR109*, *GPR43*, which the receptors may activated by SCFAs. To some extent, these results are consistent with previous studies. In our research, we further found butyric acid was positively correlated with the mRNA expression levels of *Claudin 1*, *ZO-1*, *IL-10*, and *IL-22* in the cecum mucosa, suggesting that butyric acid may play a role in boosting anti-inflammatory capacity. These results obtained are consistent with the previous work carried out by others. These results suggested that: the active state of gene expression and tight junction protein in cecum, being found in pectin treatment, may be only the basis of other biological processes. At the same time, the enhanced beneficial bacteria and increased SCFAs can provide a viable mechanism to support the accurate progress of repairing intestinal damage.

## Conclusion

In conclusion, the present study showed that dietary pectin supplementation, during the weaning period, may improve the ability of piglets to resist intestinal injury induced by LPS challenges. The addition of pectin to the diet improved the mucosal and chymous microbial disruption, and further augmented the SCFAs. Noteworthy, SCFAs improved the intestinal barrier, elevated anti-inflammatory cytokines, and reduced pro-inflammatory cytokines by activating *GPR43*, *GPR109*, and *AhR* receptors. Last, this study makes an essential contribution to the evidence base on the use of pectin in fed additives.

## Data availability statement

The datasets presented in this study can be found in online repositories. The names of the repository/repositories and accession number(s) can be found at: https://www.ncbi.nlm.nih.gov/, PRJNA889391.

## Ethics statement

The animal study was reviewed and approved by Experimental Animal Welfare and Ethical Committee of Institute of Animal Science of Chinese Academy of Agricultural Sciences.

## Author contributions

GD: animal feeding, data analyzing, and manuscript writing. WxW: conceptualization, methodology, manuscript writing, and resources. RZ and WdW: participate in the revision of manuscript. LC and HZ: conceptualization, supervision, validation, reviewing, and editing. All authors read and approved the final manuscript.

## Funding

This work was supported by National Natural Science Foundation of China (NSFC; 31802072).

## Conflict of interest

The authors declare that the research was conducted in the absence of any commercial or financial relationships that could be construed as a potential conflict of interest.

## Publisher’s note

All claims expressed in this article are solely those of the authors and do not necessarily represent those of their affiliated organizations, or those of the publisher, the editors and the reviewers. Any product that may be evaluated in this article, or claim that may be made by its manufacturer, is not guaranteed or endorsed by the publisher.
