# Supplementary figures and images for: Pectin supplement alleviates gut injury potentially through improving gut microbiota community in piglets
Source: Front Microbiol. 2022 Dec 9;13:1069694. doi: 10.3389/fmicb.2022.1069694 (PMC9780600; doi:10.3389/fmicb.2022.1069694)

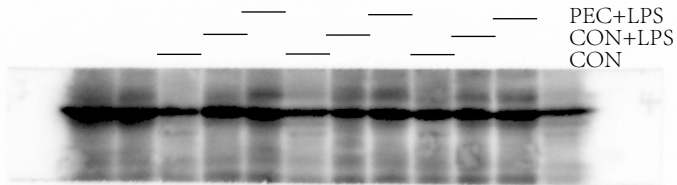

Supplement: Supplementary file 1 [file Data_Sheet_1.ZIP › β-Actin.pdf]

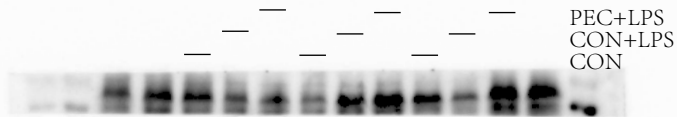

Supplement: Supplementary file 1 [file Data_Sheet_1.ZIP › claudin 1.pdf]

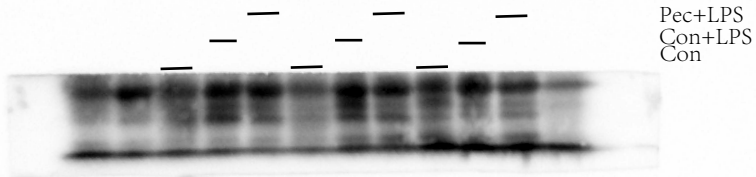

Supplement: Supplementary file 1 [file Data_Sheet_1.ZIP › Occludin1.pdf]
